# Long-term hippocampal interneuronopathy drives sex-dimorphic spatial memory impairment induced by prenatal THC exposure

**DOI:** 10.1038/s41386-020-0621-3

**Published:** 2020-01-26

**Authors:** Adán de Salas-Quiroga, Daniel García-Rincón, Daniel Gómez-Domínguez, Manuel Valero, Samuel Simón-Sánchez, Juan Paraíso-Luna, José Aguareles, Mitona Pujadas, Carolina Muguruza, Luis F. Callado, Beat Lutz, Manuel Guzmán, Liset Menéndez de la Prida, Ismael Galve-Roperh

**Affiliations:** 1 Department of Biochemistry and Molecular Biology, Instituto Ramón y Cajal de Investigación Sanitaria (IRYCIS), Instituto Universitario de Investigación Neuroquímica (IUIN), Complutense University, 28040 Madrid, Spain; 2 Centro de Investigación Biomédica en Red sobre Enfermedades Neurodegenerativas (CIBERNED), 28049 Madrid, Spain; 3 Instituto Cajal, CSIC, Avda Dr Arce 37, 28002 Madrid, Spain; 4 Integrative Pharmacology and Systems Neuroscience Research Group, Neurosciences Research Program, Hospital del Mar Medical Research Institute, Barcelona, Spain; 5 Department of Pharmacology, University of the Basque Country UPV/EHU and Centro de Investigación Biomédica en Red de Salud Mental (CIBERSAM), Leioa, Spain; 6 Institute of Physiological Chemistry, University Medical Center of the Johannes Gutenberg University Mainz, Mainz, Germany

**Keywords:** Developmental neurogenesis, Risk factors

## Abstract

Prenatal exposure to Δ^9^-tetrahydrocannabinol (THC), the most prominent active constituent of cannabis, alters neurodevelopmental plasticity with a long-term functional impact on adult offspring. Specifically, THC affects the development of pyramidal neurons and GABAergic interneurons via cannabinoid CB_1_ receptors (CB_1_R). However, the particular contribution of these two neuronal lineages to the behavioral alterations and functional deficits induced by THC is still unclear. Here, by using conditional CB_1_R knockout mice, we investigated the neurodevelopmental consequences of prenatal THC exposure in adulthood, as well as their potential sex differences. Adult mice that had been exposed to THC during embryonic development showed altered hippocampal oscillations, brain hyperexcitability, and spatial memory impairment. Remarkably, we found a clear sexual dimorphism in these effects, with males being selectively affected. At the neuronal level, we found a striking interneuronopathy of CCK-containing interneurons in the hippocampus, which was restricted to male progeny. This THC-induced CCK-interneuron reduction was not evident in mice lacking CB_1_R selectively in GABAergic interneurons, thus pointing to a cell-autonomous THC action. In vivo electrophysiological recordings of hippocampal LFPs revealed alterations in hippocampal oscillations confined to the stratum pyramidale of CA1 in male offspring. In addition, sharp-wave ripples, a major high-frequency oscillation crucial for learning and memory consolidation, were also altered, pointing to aberrant circuitries caused by persistent reduction of CCK^+^ basket cells. Taken together, these findings provide a mechanistic explanation for the long-term interneuronopathy responsible for the sex-dimorphic cognitive impairment induced by prenatal THC.

## Introduction

Exposure of the immature brain to cannabinoids exerts deleterious functional consequences in adulthood by interfering with neural cell development, as well as with neuronal differentiation and connectivity [1]. Within the more than 100 active compounds in the cannabis plant, Δ^9^-tetrahydrocannabinol (THC) is the most relevant molecule considering both its high abundance and its high potency to engage cannabinoid CB_1_ receptors (CB_1_R). The consequences of prenatal THC exposure are intrinsically different from administration in adulthood, because, during the developmental window, the pattern of CB_1_R receptor expression diverges from that in the mature nervous system, which allows to control various neural progenitor cell functions [2, 3]. Embryonic THC exposure impairs *N*-methyl-d-aspartate receptor-dependent long-term depression [4] and modulates dopamine release and receptor availability [5, 6]. Perinatal THC exposure also induces changes in glutamatergic and noradrenergic signaling that may contribute to the cognitive deficits observed in adulthood [7, 8]. The consequences of cannabinoid exposure during the highly vulnerable adolescence period have been the subject of intense research [9]. In adolescence, THC induces a subcortical hyperdopaminergic state and adaptations in the prefrontal cortex, and these plasticity changes are associated to decreased social interaction, higher anxiety, and altered sensorimotor gating [10]. In contrast, in the adult brain, THC blunts dopaminergic function and is associated with negative emotionality and addiction severity [11]. Similarly, maternal cannabis consumption results in a selective impairment of D_2_R-dependent (but not D_1_R-dependent) dopaminergic neurotransmission in the developing mesolimbic system [6, 12, 13].

THC interferes with the delicate neurodevelopmental role of the endocannabinoid system [2, 3]. CB_1_R, upon engagement by endocannabinoids (2-arachidonoylglycerol and anandamide), regulates crucial steps of cortical development, including neural progenitor proliferation, neuronal differentiation, and neuronal migration. Hence, pharmacological or genetic manipulation of CB_1_R function in the embryonic brain results in long-lasting alterations. Specifically, prenatal cannabinoid signaling regulates interneuron migration and morphogenesis [14], and administration of the CB_1_R/CB_2_R-mixed agonist WIN-55,212-2 selectively reduces hippocampal cholecystokinin (CCK) basket cell (BC) neurotransmission, without altering pyramidal neurons [15]. Upon prenatal exposure to WIN-55,212-2, mice exhibit reduced depolarization-induced suppression of inhibition and feedforward inhibition, as well as altered social interaction. However, embryonic THC administration can also interfere with pyramidal neuron development, such as deep-layer (Vb) corticospinal motor neurons [16]. These changes are associated to an unbalanced excitation/inhibition state, thus resulting in a higher susceptibility to seizures. Studies based on conditional CB_1_R genetic rescue from a CB_1_R-null background have demonstrated a cell-autonomous action of THC in dorsal telencephalic principal neurons associated to skilled motor impairment, while a proconvulsive THC-induced status relies, at least in part, on alterations of both pyramidal neurons and GABAergic interneurons [16]. Similarly, prenatal administration of WIN-55,212-2 exerts alterations in pyramidal neuron excitability of the prefrontal cortex in adult male rats [17]. In the present study, evaluation of the neurodevelopmental impact of prenatal cannabinoid exposure revealed a striking sex-dependent impact on hippocampal plasticity, with males being selectively affected. Hence, in the context of the progressive worldwide legalization of cannabis use for both medical and recreational purposes [18], this study strongly supports that the functional consequences of prenatal THC exposure should be assessed considering the existence of sex-dependent differences in both intensity and nature of THC-induced alterations.

## Materials and methods

Detailed methods for immunofluorescence and confocal microscopy, in situ hybridization (ISH), immunohistochemistry (IHC), stereological analysis, pentylenetetrazol (PTZ)-induced seizures assay, novel object recognition (NOR) and object location (OL) tasks, cannabinoid-induced analgesia and hypothermia, THC measurements, and WIN-55,212-2-stimulated [^35^S]GTPγS-binding assays are provided as Supplementary Materials and Methods.

### Animals

Experimental designs and procedures were approved by the Complutense University Animal Research Committee in accordance with the European Commission regulations. The generation and genotyping of Nex-CB_1_^−/−^, Dlx5/6-CB_1_^−/−^, and control CB_1_^f/f^ littermates has been reported elsewhere [19]. Neuronal population changes and CB_1_R status were analyzed at different time points (P20 and P90) after electrophysiological characterization and behavioral determinations performed at P60.

### In vivo electrophysiological recordings

Mice were implanted with chamber/fixation bars under isoflurane anesthesia (1.5–2% mixed in oxygen 400–800 ml/min). The recording chamber was aligned to target the right dorsal hippocampus at −1.8 mm posterior from the bregma and 1.25 mm lateral from midline. At least two jeweller’s screws were inserted into the skull for providing additional anchoring and reference/ground connections (over the cerebellum). An intracerebellar silver wire was used as the main reference/ground connection. The implant was secured with dental cement. Animals were recovered from anesthesia and were returned to home cages.

After surgery, mice were habituated to the head-fixed setup consisting on a cylinder (20 cm radius) coupled to a stereotactic frame. Habituation sessions included handling, running freely around the setup, and mounting/dismounting the head during brief periods of time. After 2–3 days, animals were water-deprived and they started daily sessions of 10 min running for reward (water), till they were fully habituated. We increased the time in the setup over steps of 5 min, till animals got habituated to behave freely in the cylinder. They typically alternated periods of running and immobility during a maximum of 60 min each day. Water port was removed after the first 10 min session to keep deprivation level. Once mice were habituated to a 30–60 min session, they were anesthetized to open the craniotomy. Afterwards, craniotomy was covered with low toxicity silicone elastomer (Kwik-Sil^TM^, World Precision Instruments) and recordings started the day after. Every animal was recorded in two independent sessions of 1 h.

For recordings, we used 16-channel silicon probes consisting in a linear (100 µm resolution, 413 µm^2^ electrode area, Neuronexus). Extracellular signals were pre-amplified (4x gain) and recorded with a 16-channel AC amplifier (Multichannel Systems), further amplified by 100, filtered by analog means at 1 Hz to 5 kHz, and sampled at 20 kHz/channel with 12 bits precision. The animal speed was stored to evaluate periods of running and immobility.

Analysis of electrophysiological signals was implemented in MATLAB 9.3 (MathWorks). Local-field potential (LFP) signals from sites at *stratum lacunosum moleculare* (*slm*) were used for identifying *θ* periods during running (bandpass 4–12 Hz). Forward–backward zero-phase finite impulse response filters of order 512 were used to preserve temporal relationships between channels and signals. The mean power spectra during *θ* was fitted to the 1/*f* decay for frequencies >60 Hz and a reference level was established at 0 dB. Spectral values fitted to 1/*f* were similar between groups and were discarded for the analysis. Consequently, only the *θ* (4–12 Hz) and low *γ* bands (30–60 Hz) were included in the analysis.

For detection of sharp-wave ripple (SWR), LFP signals from *stratum* *radiatum* (*sr*) were low-pass filtered (100 Hz), whereas signals from *stratum pyramidale* (*sp*) were bandpass filtered (100–600 Hz). Filtered signals were smoothed by a Gaussian kernel and candidate events were detected by thresholding (>4 SDs). The power spectra was evaluated in a window of ±0.2 ms around each detected event. Time–frequency analysis was performed by applying the multi-taper spectral estimation in sliding windows with 97.7% overlap and frequency resolution of 10 Hz in the 90–600 Hz frequency range. The normalized power in the 90–600 Hz band was treated as a statistical distribution, as previously described [20]. Slow (90–120 Hz) and fast ripples (>120 Hz) were separated using individual spectra for visualization purposes.

### Data and statistical analyses

Results shown represent the means ± SEM and the number of experiments is indicated in every case. Statistical analysis was performed with GraphPad Prism 6.07 (GraphPad Software, La Jolla, CA, USA). All variables were first tested for normality (Kolmogorov–Smirnov) and homoscedasticity (Levene’s). When variables satisfied these conditions, two-way analysis of variance and Fisher’s ﻿least significance difference *post hoc* test were used to assess differences between groups. *P*-values < 0.05 were regarded as statistically significant.

## Results

### Embryonic THC exposure evokes long-term interneuron alterations in a sex-dimorphic manner

**Fig. 1:**
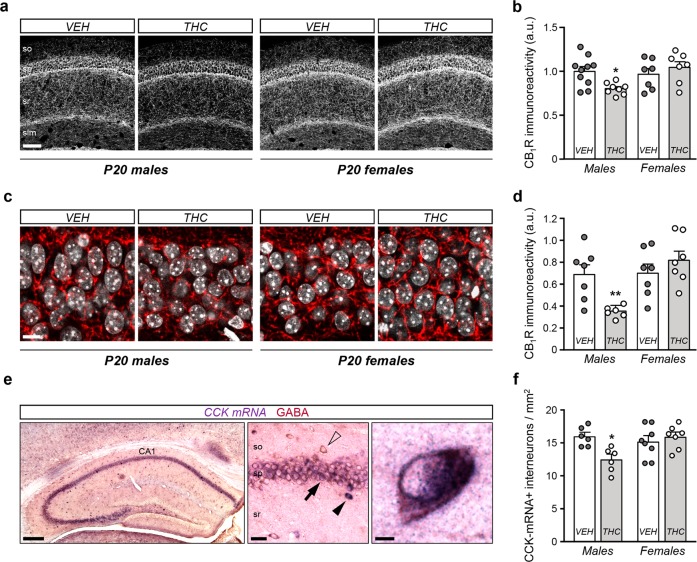
Prenatal THC exposure induces a sex-dimorphic long-lasting hippocampal CB_1_R adaptation and CCK^+^ basket cell interneuronopathy. **a**, **b** Representative images and quantification of CB_1_R immunoreactivity measured in the CA1 hippocampal region of P20 mice prenatally exposed to THC (3 mg/kg) or its vehicle. **c**, **d** CB_1_R immunoreactivity quantified in the stratum pyramidale of CA1 in the same mice. **e**, **f** Representative images of CCK in situ hybridization and GABA immunohistochemistry in the hippocampus, and quantification of interneurons identified as CCK/GABA double-positive cells. *n* *=* at least 7 (**a**) or 6 (**d**, **f**) animals per group. *p < 0.05 vs. corresponding vehicle; **p < 0.01 vs. corresponding vehicle. Scale bars: **a** 50 µm; **c** 10 µm; **e** left 150 µm, center 25 µm, right 5 µm.

To assess the impact of prenatal THC administration throughout interneuron embryonic development, we defined a temporal window of drug delivery at 3 mg/kg (intraperitoneally) from E10.5 to E17.5. At P20, analysis of CB_1_R immunoreactivity in the dorsal hippocampus of wild-type mice prenatally exposed to THC showed a male-specific decrease of CB_1_R levels with respect to vehicle-treated mice (Fig. 1a, b and Supplementary Fig. S1a, b). Interestingly, no significant differences were found for sex or treatment in other areas as the prefrontal or somatosensory cortex (Supplementary Fig. S1c, d). Of note, the vast majority of immunoreactivity that is detected under these experimental conditions corresponds to CB_1_R^+^ terminals of GABAergic interneurons [16, 21]. Hence, we decided to focus on CA1 CCK^+^ BCs, the population of hippocampal interneurons that expresses the highest levels of CB_1_R in the adult mouse forebrain [22]. Perisomatic CB_1_R immunoreactivity at CA1 *sp* was reduced in the THC-exposed male progeny compared with its vehicle-treated counterpart (Fig. 1c, d). No differences were found for females treated with either THC or its vehicle.

Next, we evaluated whether these alterations are due to a previously described morphogenetic role of CB_1_R in developing interneurons [23] or to a decrease in the total number of CCK^+^ interneurons. To address this question, we performed ISH with a riboprobe against CCK, combined with anti-GABA IHC to unequivocally identify CCK-containing hippocampal interneurons, given the broad expression of CCK by CA1 pyramidal neurons (Fig. 1e and Supplementary Fig. S1e). Stereological analysis showed a selective reduction of CA1 CCK^+^ interneuron density in THC-treated males as compared either with vehicle-treated males or with females irrespective of the treatment (Fig. 1f). Taken together, these findings support that those mice that had been exposed to THC during embryonic development exhibit an overt interneuronopathy with a remarkable sexual dimorphism. To rule out the possibility that sexual differences emerge from pharmacokinetic factors, such as THC bioavailability, we measured THC levels in E17.5-brain tissue (i.e., 12 h after the last THC injection) and found similar values in both sexes (Supplementary Fig. S1f). In addition, as protracted embryonic THC exposure leads to a transient downregulation of CB_1_R [16], we asked whether a sex-dependent CB_1_R downregulation might be responsible for the observed effects. [^35^S]GTPƳS-binding analysis in E17.5-brain tissue revealed an evident downregulation of CB_1_R after embryonic THC exposure, but comparable curves were obtained for both sexes (Supplementary Fig. S1g).

### Prenatal THC exposure alters main hippocampal oscillations in adult males

**Fig. 2:**
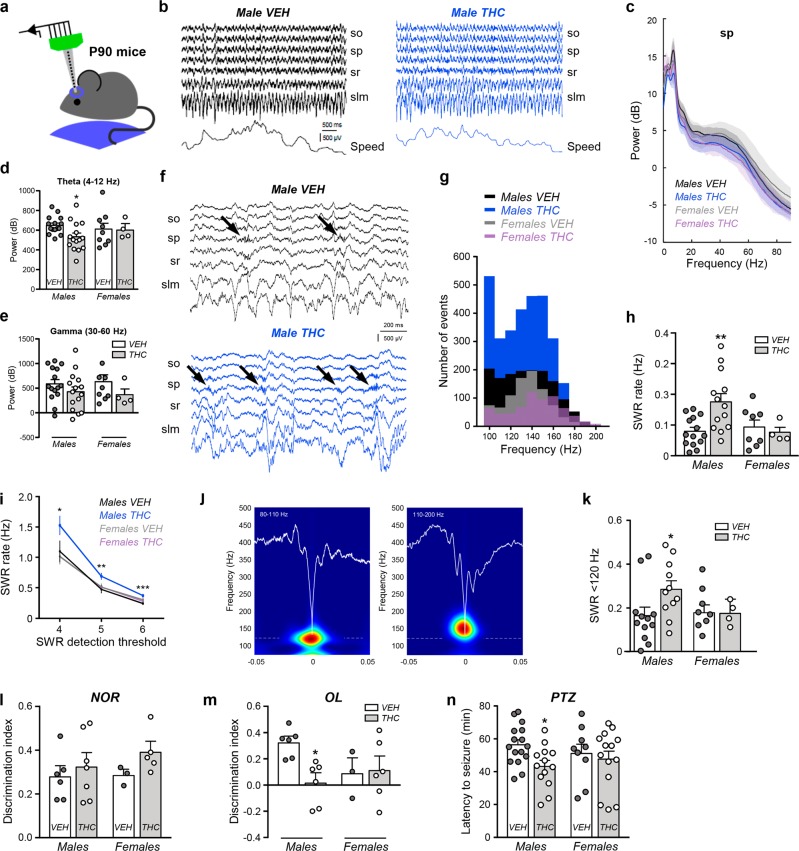
Prenatal THC exposure induces sex-dimorphic alterations of hippocampal oscillations and spatial cognitive deficits. **a** Scheme of experimental setup. P60-P90 mice were recorded head-fixed while running freely on a wheel. **b** Laminar recordings of LFP signals from a representative male mouse from each group (vehicle and THC). The running speed is shown at the bottom. so, stratum oriens; sp, stratum pyramidale; sr, stratum radiatum; slm, stratum lacunosum moleculare. **c** Mean power spectra of running episodes show representative peaks at *θ* and *γ* recorded at the pyramidal cell layer (*sp*). *n* = 15 vehicle-treated males, 15 THC-treated males, 9 vehicle-treated females, 4 THC-treated females. **d** Group differences of *θ* power were specific for male THC mice. *p < 0.05. Same dataset as above. **e** No differences of *γ* power. **f** SWR events were recorded during immobility. Note higher event rate in THC-treated males (arrows). **g** Distribution of spectral peaks per SWR events in the different groups. Note higher number of events in males THC and a larger contribution of slow ripple events. **h** Group differences of SWR rate, **p < 0.01. **i** Consistent larger SWR rate across detection thresholds (in number of SD). **j** Examples of slow (80–110 Hz) and fast (110–200 Hz) SRW events. **k** The proportion of slow SWR was significantly higher in the male THC group. Same dataset as above. *p < 0.05. **l** Mean group values of discrimination index in the NOR task. *n* = 6 vehicle-treated males, 7 THC-treated males, 3 vehicle-treated females, 5 THC-treated females. **m** Mean group values of discrimination index in the OL task. Same dataset as NOR task. **n** Latency to PTZ-induced seizures was significantly lower in the male THC group. *p < 0.05 *n* = 16 vehicle-treated males, 13 THC-treated males, 9 vehicle-treated females, 14 THC-treated females.

CCK^+^ BCs are essential for proper hippocampal physiology and function [24–26]. Hence, we decided to further characterize the aforementioned THC-induced deficits via intrahippocampal recordings. Specifically, we performed in vivo electrophysiological recordings of the LFP throughout CA1 in the hippocampus of head-fixed P60 adult mice (Fig. 2a). During running, *θ* (4–12 Hz) and *γ* oscillations (30–60 Hz) typical of exploratory behavior were recorded across layers (Fig. 2b). Spectral analyses of the LFP signal revealed changes in the *θ* band that were confined to the *sp* of CA1 (Fig. 2c, d and Supplementary Fig. S2a, b). THC treatment tended to reduce the *γ* power at *sp* consistently in both males and females (Fig. 2e). These data align with the idea of a complementary role of CCK BCs in hippocampal *θ* and *γ* oscillations [25], and the impact of THC administration in network oscillations and psychiatric traits [27].

Next, we analyzed SWRs (Fig. 2f), a major hippocampal high-frequency oscillation recorded during immobility and sleep that is crucial for learning and memory consolidation [28]. SWR emerge from the oscillatory firing of pyramidal cell ensembles, controlled by the interplay between excitation and BC-mediated inhibition [29]. In diseases affecting the hippocampal formation such as temporal lobe epilepsy and Alzheimer’s disease, SWR are pathological and associated to severe cognitive deficits and epileptiform activities [30, 31]. Spectral analysis of SWR in mice treated with THC confirmed some differences with a clear sex selectivity (Fig. 2g). First, the SWR rate was significantly higher in males exposed to THC as compared with females or vehicle-treated males, independently of the detection threshold (Fig. 2g–i). Second, the typical bimodality of the spectral SWR peaks [32] was remarked in THC-treated male mice (Fig. 2g), with many more slower (<120 Hz) than fast events (>120 Hz) (Fig. 2j). This reflects a larger proportion of high-*γ* SWR in THC-exposed males as compared with the other groups (Fig. 2k and Supplementary Fig. S2f). No significant changes were found in other measures of spectral distribution such as entropy and fast ripple index (Supplementary Fig. S2c–e). Taken together, these observations provide functional evidence for aberrant hippocampal microcircuit function caused by a persistent reduction of CCK^+^ interneurons and oscillatory defects, which predict cognitive alterations in males prenatally exposed to THC. To test this idea further, we conducted NOR and OL memory tests. The former evaluates non-spatial, conceptual learning of object identity, which depends on multiple brain regions, while the latter provides a measure of spatial learning, which strongly relies on hippocampal function [33]. We did not observe any interference of prenatal THC in the NOR paradigm, but THC-treated males performed poorly in the OL test as compared with vehicle-treated counterparts (Fig. 2l–m and Supplementary Fig. S3a–c), consistent with the aforementioned electrophysiological data. In addition, we analyzed susceptibility to PTZ-induced seizures and found selective effects in the male offspring (Fig. 2n). Altogether, these data support deleterious sex-dimorphic effects of prenatal THC exposure caused by a CCK interneuronopathy.

### CB_1_R located on GABAergic neurons is responsible for the sex-dimorphic interneuronopathy induced by embryonic THC exposure

**Fig. 3:**
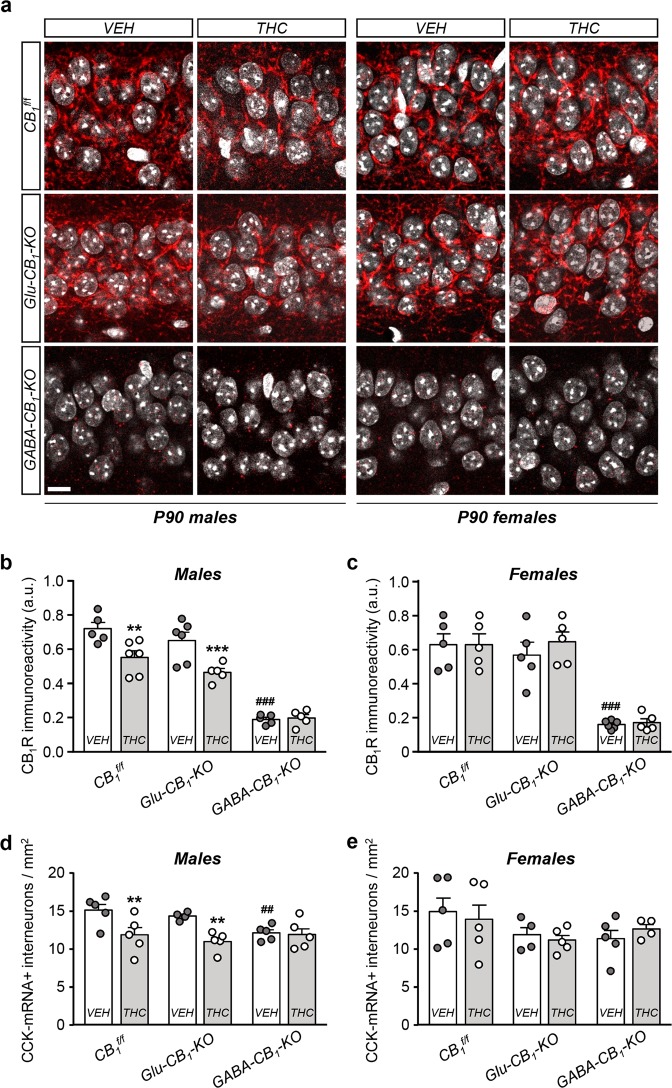
Sex-specific interneuronopathy elicited by prenatal THC exposure requires CB_1_R located on GABAergic neurons. **a** Representative images of CB_1_R immunofluorescence in the *sp* of the CA1 hippocampal region of *Glu-CB*_*1*_*-KO*, *GABA-CB*_*1*_*-KO* and *CB*_*1*_^*f/f*^ P90 mice prenatally exposed to THC or its vehicle. **b**, **c** Quantification of CB_1_R immunoreactivity in the area shown above both in males and females. **d**, **e** Quantification of CCK^+^ interneurons in the same animal groups segregated in males and females. *n* = at least 5 animals per group. **p < 0.05 vs. corresponding vehicle; ***p < 0.001 vs. corresponding vehicle; ^##^*p* < 0.01 vs. *CB1*^*f/f*^ vehicle group; ^###^*p* < 0.001 vs. *CB1*^*f/f*^ vehicle group. Scale bar: 10 µm.

To understand in further detail the developmental mechanisms leading to CCK interneuronopathy in adulthood, we sought for a direct link between THC action and specific cellular targets. CB_1_R is expressed in various neuronal lineages throughout development [3]. To unequivocally assess the involvement of a specific subset of CB_1_R in the abovementioned defects, we made use of conditional knockout mice lacking CB_1_R exclusively in dorsal telencephalic glutamatergic pyramidal cells (*CB*_*1*_^*floxed/floxed;Nex-Cre/+*^ mice; herein referred to as *Glu-CB*_*1*_*-KO*) or in forebrain GABAergic neurons (*CB*_*1*_^*floxed/floxed;Dlx5/6-Cre/+*^ mice; herein referred to as *GABA-CB*_*1*_*-KO*) [21]. Immunofluorescence analysis of perisomatic CB_1_R^+^ BC synapses at CA1 *sp* revealed a selective long-term decrease in *CB*_*1*_^*f/f*^ males exposed to THC, which was preserved in *Glu-CB*_*1*_*-KO* animals, pointing to an involvement of CB_1_R located on developing GABAergic interneurons in mediating THC actions (Fig. 3a–c). In contrast, the remnant hippocampal CB_1_R immunoreactivity of *GABA-CB*_*1*_*-KO* mice exhibited no differences by sex or treatment (Fig. 3a–c). In addition, we carried out anti-CCK ISH combined with anti-GABA IHC to label CCK^+^ hippocampal interneurons in every genotype, sex, and treatment. Stereological analysis confirmed a persistent reduction of CCK^+^ interneurons in *CB*_*1*_^*f/f*^ males prenatally exposed to THC compared with vehicle-treated littermates (Fig. 3d), whereas *CB*_*1*_^*f/f*^ females exhibited comparable densities irrespective of treatment (Fig. 3e). Likewise, similar data were obtained in *Glu-CB*_*1*_*-KO* mice, whereas *GABA-CB*_*1*_*-KO* mice appeared refractory to THC impact in both males and females (Fig. 3d, e). Remarkably, conditional deletion of CB_1_R in the GABAergic lineage *per se* led to a decrease in the density of CCK^+^ hippocampal interneurons, only reaching statistical significance in the male population (Fig. 3d). These findings demonstrate the involvement of CB_1_R located on hippocampal GABAergic interneurons as the main target for the sex-dimorphic impact of embryonic THC exposure.

### Cell population-specific sex dimorphism of CB_1_R signaling is critical for adult hippocampal function

**Fig. 4:**
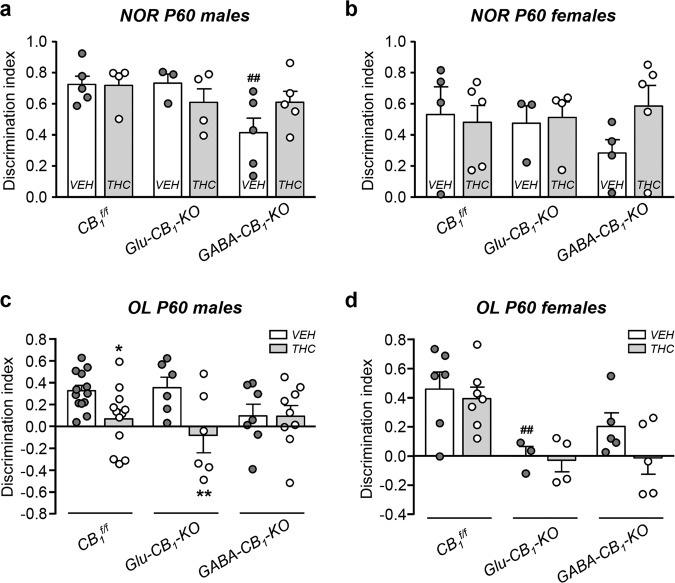
Hippocampal-dependent spatial memory, but no object recognition memory, is altered by prenatal THC exposure specifically in males that preserve CB_1_R in GABAergic neurons. **a**, **b** Novel object recognition test was performed in *Glu-CB*_*1*_*-KO*, *GABA-CB*_*1*_*-KO*, and *CB*_*1*_^*f/f*^ P60 mice, and a discrimination index was calculated for both males and females. **c**, **d** Object location test was performed in the mentioned groups, a similar index was then calculated for males and females. *n* *=* 3–14 animals per group. *p < 0.05 vs. corresponding vehicle; **p < 0.01 vs. corresponding vehicle; ^##^*p* < 0.01 vs. *CB*_*1*_^*f/f*^ vehicle group.

**Fig. 5:**
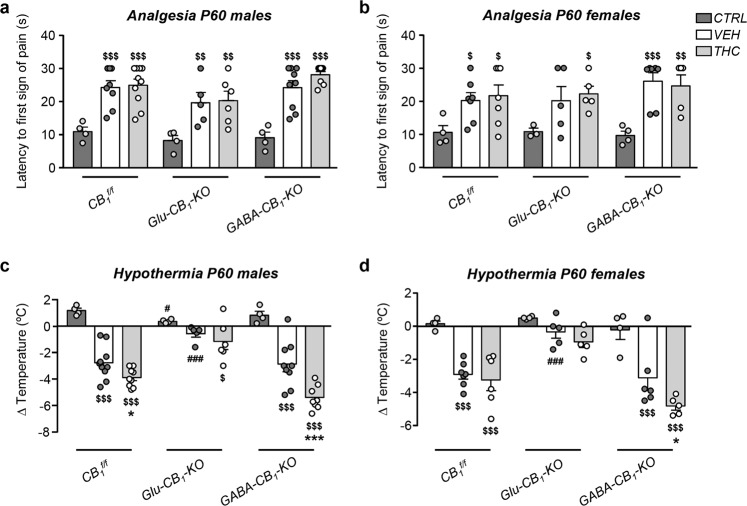
THC-induced actions that depend on principal neurons are not sex-dependent. **a**, **b** THC-induced analgesia was analyzed in vehicle- and THC-treated *Glu-CB*_*1*_*-KO*, *GABA-CB*_*1*_*-KO*, and *CB*_*1*_^*f/f*^ P60 male and female mice. **c**, **d** THC-induced hypothermia was analyzed in the same animal groups. *n* *=* 3–14 animals per group. *p < 0.05 vs. corresponding vehicle; ***p < 0.001 vs. corresponding vehicle; ^#^*p* < 0.05 vs. *CB1*^*f/f*^ vehicle group; ^###^*p* < 0.001 vs. *CB1*^*f/f*^ vehicle group; ^$^*p* < 0.05 vs. control group within each genotype; ^$$^*p* < 0.01 vs. control group within each genotype; ^$$$^*p* < 0.001 vs. control group within each genotype.

To unequivocally identify the neuronal population responsible for the prenatal THC-induced cognitive impairment, we performed the NOR and OL tests in conditional CB_1_R-deficient mice. Prenatal THC administration did not induce significant differences in the NOR task by sex or treatment in *CB*_*1*_^*f/f*^ and *Glu-CB*_*1*_*-KO* mice (Fig. 4a, b and Supplementary Fig. S3d–i). In contrast, we found a robust spatial memory impairment in THC-treated *CB1*^*f/f*^ and *Glu-CB*_*1*_*-KO* male mice in the OL task (Fig. 4c and Supplementary Fig. S3j–o). THC exerted no effect in *GABA-CB*_*1*_*-KO* males, which exhibited worse spatial memory as compared with vehicle-treated *CB*_*1*_^*f/f*^ and *Glu-CB*_*1*_*-KO* males (Fig. 4c). Surprisingly, a different mechanism seems to operate in females, which exhibited an interaction between genotype and treatment (Fig. 4d), hence suggesting that additional CB_1_R neuron populations may contribute to spatial cognition. Interestingly, vehicle-treated *GABA-CB*_*1*_*-KO* male mice presented impairment of conceptual memory, which was not evident upon embryonic THC administration (Fig. 4a, b). In this regard, NOR performance is known to be affected in *GABA-CB*_*1*_*-KO* mice [34]. Together, these data support a requirement of CB_1_R signaling in developing GABAergic interneurons for the adequate maturation of CCK-dependent hippocampal function in male mice, and point to remarkable sex-dimorphic actions of CB_1_R signaling along development. To further assess the specific involvement of CB_1_R located on GABAergic interneurons in the developmental consequences of THC exposure, we analyzed the impact of THC on two behavioral traits that depend mainly on CB_1_R located on principal neurons. Thus, no genotype or sex interaction was observed in THC-induced analgesia, a trait that relies on principal neurons located outside the neocortex [19] (Fig. 5a, b). Moreover, cannabinoid-induced hypothermia, which is mediated mainly by CB_1_R on dorsal telencephalic glutamatergic neurons [19], did not reveal sex or treatment interaction in *Glu-CB*_*1*_*-KO* mice, but showed treatment interaction and no sex dimorphism in *GABA-CB*_*1*_*-KO* animals (Fig. 5c, d).

## Discussion

The demonstration of a functional role of endocannabinoid signaling in modulating crucial neurodevelopmental processes [2, 3, 35] has contributed to the understanding of the consequences of prenatal cannabinoid exposure in developing neuronal circuits [12]. Here we demonstrate that prenatal THC exposure exerts a sex-dependent interference with CCK BC development, thereby leading to long-term interneuronopathy, altered hippocampal function, and impaired spatial cognition. Several studies have investigated the consequences of prenatal exposure to cannabinoids on GABAergic interneuron development. Our results are in partial agreement with previous findings showing that prenatal WIN-55,212-2 administration interferes with the development of CCK BCs, which in turn impacts feedforward and feedback inhibition [15]. This effect was associated with reduced social interaction, but not with anxiety. Likewise, WIN-55,212-2 administration during early and mid, but not at late, adolescence interferes with maturation of GABAergic function, leading to layer V prefrontal cortex disinhibition [36]. Hence, both perinatal and adolescent exposure to cannabinoids result in GABAergic hypofunction. The contribution of this GABAergic hypofunction to the neuropsychiatric traits induced by developmental cannabinoid exposure has been mainly associated to increased risk of developing psychosis or schizophrenia [37]. Alternatively, cannabinoid-induced remodeling and plasticity of GABAergic circuits can contribute to cognitive impairment [38]. CB_1_R signaling is critical in cognition and memory, and hence cannabinoid exposure interferes with working and episodic memory [38]. Although in the adult brain acute and chronic THC administration impairs NOR in males and females [34], our results indicate that embryonic THC exposure does not affect conceptual learning, but instead blunts spatial memory, and this occurs in a striking sex-dependent manner.

Our results also show a reduction of CCK interneurons and a selective impairment of spatial memory, consistent with the major role of hippocampal CCK BCs in spatial information coding and control of hippocampal oscillations [25, 39]. A reduction in both CCK BC density and inhibitory tone decreases the power of *θ* oscillations during exploratory behavior and disrupts place cell-dependent spatial coding, hence impairing spatial learning [39]. We found changes in the *θ* and the low ripple band exclusively in THC-treated male mice. Strikingly, *θ*-nested *γ* oscillations were not affected, thus confirming frequency- and circuit-specific effects. CB_1_R differentially contributes to perisomatic inhibition of superficial vs. deep CA1 pyramidal cells [26]. Hence, given the ability of CCK interneurons that express CB_1_R to influence SWR [26], and the role of SWR in memory [40], our findings support the notion that disrupted hippocampal oscillations are responsible for particular forms of cognitive impairment induced by prenatal THC exposure. In adult mice, acute THC-induced alterations in synchronized neural oscillations in the *γ* (30–80 Hz) and *θ* (4–7 Hz) ranges have been linked to psychosis-related alterations [27], and CB_1_R-dependent regulation of cortical and subcortical network synchrony has been proposed to participate on THC-induced alterations of sensory perception [41]. In this regard, a thorough characterization of hippocampal oscillatory activity in mice lacking CB_1_R selectively in interneurons or principal neurons would shed additional light on the precise functional contribution and sensitivity to prenatal THC exposure of different CB_1_R-expressing neuronal populations to network activity and cognitive function, an issue that deserves future research. Previous findings suggest that CB_1_R controls spatial memory by regulating hyperpolarization-activated cyclic nucleotide-gated (HCN) cationic channels and current Ih, an effect that is specific of CA1 superficial pyramidal neurons [42]. Possibly, the frequency-specific effect found in ripple distribution of THC-treated males can be associated with the emerging concept of different microcircuit organization along the deep-superficial hippocampal sublayers [43, 44]. The immediate early gene transcription factor NPAS4 mediates experience-driven recruitment of CCK-evoked cannabinoid inhibition [45]. Hence, enriched environment may constitute a valuable strategy to counteract the detrimental consequences of prenatal cannabinoid exposure as an alternative to ongoing studies aimed to prevent deleterious cannabinoid maladaptive plasticity by pharmacological manipulation [13, 46].

Our findings highlight the importance of addressing sex differences when investigating the neurodevelopmental changes induced by cannabis exposure and the functional consequences in the offspring [47]. Males and females possess different expression levels of endocannabinoid system elements, respond differently to THC, and hence are differently affected in various cannabinoid-related parameters [48, 49]. In addition, whereas we cannot completely exclude that changes of oestrous cycle may play a role in the behavioral differences induced by prenatal THC exposure, the male-selective observed interneuronopathy makes this possibility very unlikely. In animal models, prenatal cannabinoid exposure induces sex-dimorphic changes in pyramidal neuron intrinsic properties and synaptic plasticity in the PFC, which are in turn associated to social interaction deficits [17]. In addition, sex-dimorphic THC-induced CCK BC interneuronopathy contributes to spatial cognitive impairment (present study) and THC-induced hyperdopaminergic state is also male selective [13]. The existence of a sex-dependent bias in cannabinoid-induced interference with social interaction mediated by a loss of GABAergic perisomatic inhibition remains unknown [15]. The nature of this bias would thus require further investigation. Indeed, brain wide-mapping studies have recently revealed cell-type-specific contributions to cortical and subcortical sexual dimorphism [50], suggesting that many effects should be identified at the system level.

In our hands, we did not find that embryonic THC influences psychotic-like features in the offspring (startle response and prepulse inhibition; data not shown). One possible explanation is that, when modeling human cannabinoid exposure in laboratory animals, WIN-55,212-2 is likely to induce a stronger impact than THC and other phytocannabinoid molecules. Phytocannabinoids possess important differences in solubility, potency, and hence pharmacokinetic and pharmacodynamic behavior, compared with synthetic cannabinoid drugs used for research purposes (e.g., WIN-55,212-2, HU-210). Moreover, different cannabinoid ligands contribute differentially to biased CB_1_R signaling and can target additional receptors and binding proteins. Noteworthy, a severe cannabinoid-induced impairment of cortical oscillations was observed upon administration of the WIN-55,212-2 compound [36, 51]. Hence, it would be desirable that, when attempting to extrapolate to humans the consequences of cannabinoid exposure from experimental models based on small laboratory animals, exquisite care is taken in the pharmacological regulation strategy and the experimental design that is used. Evidence for the impact of sex-dimorphic prenatal cannabinoid exposure on human brain development is scarce and its interpretation is extremely complex due to a wide array of confounding factors. Nonetheless, early human cannabinoid exposure contributes differently to drug addiction vulnerability [52] and the development of aggressiveness in male and female offspring [53].

Here we demonstrate that conditional CB_1_R ablation in the interneuron lineage induces a similar sex-dependent interneuronopathy and spatial memory impairment as that evoked by prenatal THC treatment, thus pointing to CB_1_R receptor loss of function as the main mechanism of THC action on the analyzed traits. Overall, repeated cannabinoid exposure in the immature brain results in functional antagonism of CB_1_R signaling in principal neurons [16] and CCK BCs (present study). As a consequence of THC treatment, CB_1_R desensitizes and the resulting cannabinoid signaling impairment would favor the development of seizures and spatial cognitive deficits in a sex-dimorphic manner. In agreement, post-hoc sex-dependent analyses of PTZ-induced seizure susceptibility in mice conditionally CB_1_R-rescued from a CB_1_R-null background and subjected to prenatal THC treatment [16] confirms the involvement of GABAergic interneurons, but not principal neurons, in the THC-induced hyperexcitability of the male progeny (Supplementary Fig. S4). Recently, some consequences of prenatal THC exposure in the male offspring have been shown to be rescued by co-administration of pregnenolone, a CB_1_R modulator [13]. In summary, the study of functional consequences (hyperexcitability, cognition, and other behavioral traits), in vivo electrophysiological recordings, and neuron-lineage tracking in conditional CB_1_R-deficient mice, as shown herein, demonstrates that neurodevelopmental exposure to THC exerts a sex-dependent interneuronopathy that selectively affects spatial cognitive function in male offspring.

## Funding and disclosure

The authors declare no conflict of interest. This work was supported by grants PI18-00941 to IG-R cofinanced by the European Development Regional Fund “A way to achieve Europe”; RTI2018-095311-B-100 to MG, BFU2015-66887-R to LM-P, and 2017-SGR-138 to MP from the Generalitat de Catalunya. DG-R was supported by Fundación Tatiana Pérez de Guzmán; DG-D was supported by a PhD fellowship from the Spanish Ministry of Economy and Competitiveness (BES-2013-064171). JP-L and JA were supported by FPI and FPU program fellowships, respectively (Ministerio de Educación, Cultura y Deporte) and S. S-S. was supported by Fondo Social Europeo-YEI (CT101/18-CT102/18PEJD-2018-PRE/BMD-7933). CM is recipient of a Marie Curie program fellowship (747487).

## Supplementary information

Supp Material Fig legends

Supplemental Material 1

Supplemental Material 2

Supplemental Material 3

Supplemental Material 4

Supp Methods
