# Supplementary material for: Long-term hippocampal interneuronopathy drives sex-dimorphic spatial memory impairment induced by prenatal THC exposure
Source: Neuropsychopharmacology. 2020 Jan 26;45(5):877–86. doi: 10.1038/s41386-020-0621-3 (PMC7075920; doi:10.1038/s41386-020-0621-3)
Supplement: Supplementary file 1 — Supp Material Fig legends [file 41386_2020_621_MOESM1_ESM.docx]

**Supplementary Figure Legends**

**Supplementary Fig. S1.** **Sex divergent and long-lasting CB_1_R expression plasticity affects selectively hippocampal region.** (a-d) CB_1_R immunoreactivity quantification in the progeny of vehicle- or THC- treated pregnant dams. CB_1_R levels were analyzed in the whole hippocampus (a), dentate gyrus (b), prefrontal (c) and somatosensory (d) cortices. *n=* 5 animals per group. (e) Hippocampal formation stained by CCK *in situ* hybridization without GABA immunohistochemistry. Inset: positive cells outside *stratum pyramidale* (arrowhead) can be considered as CCK^+^ basket cells safely, whereas positive cells inside *stratum pyramidale* (arrow) may be basket or pyramidal cells, needing an additional interneuronal marker as GABA to be fully identified. (f) THC levels were determined in brain extracts from E17 embryos after prenatal exposure to THC or its vehicle. *n= 6* animals per group. (g) [^35^S]GTPγS binding was determined in brain membrane extracts obtained from the same embryo cohorts. *n =* 6 pooled samples per group, each pool consisting of 3 E17.5 brains. *, p < 0.05 *versus* corresponding vehicle; **, p < 0.01 *versus* corresponding vehicle. Scale bars: 150 µm; inset: 25 µm.

**Supplementary Fig. S2. Analysis of hippocampal oscillations.** (a) Mean power spectra of running episodes recorded at SR (left) with mean group values for the theta and gamma band (right). (b) Mean power spectra of running episodes recorded at SLM (left) with mean group values for the theta and gamma band (right). (c) Distribution of the spectral entropy and fast ripple index of all SWR events recorded from different groups. (d) Mean group histograms of spectral entropy of SWR show no overt differences. (e) Same for fast ripple index. (f) Mean traces of slow and fast SWR recorded from representative mice from each group.

**Supplementary Fig. S3.** **Additional behavior parameters assessed in NOR and OL experiments.** (a, b) Total exploration time for the animals analyzed in NOR and OL tasks represented in Figure 2l and 2m, respectively. (c) Distance traveled by the same animals in an ActiTrack setup. (d – i) Total exploration time, distance traveled and global activity measured for males (d, e and f, respectively) and females (g, h, I, respectively) included in the NOR task represented in Figure 4a and 4b. (j – o). Total exploration time, distance traveled and global activity measured for males (j, k and l, respectively) and females (m, n, o, respectively) included in the OL task represented in Figure 4c and 4d.

**Supplementary Fig. S4. Seizure susceptibility in CB_1_R rescue mice.** (a-b) Seizure susceptibility to subconvulsive doses of PTZ was determined in the offspring of mice that had been prenatally exposed to vehicle or THC [data taken from their original source ^16^ and herein just segregated in males and females]. Latency to seizures was determined in CB_1_-RS, GABA-CB_1_-RS and Glu-CB_1_-RS, which express CB_1_R globally, in the GABAergic neuronal lineage, and in the glutamatergic neuronal lineage, respectively. males *n* = 9 and 9, vehicle- and THC-treated CB₁-RS, respectively; 12 and 7, vehicle- and THC-treated CB_1_-STOP, respectively; 4 and 6, vehicle- and THC-treated GABA-CB₁-RS, respectively; 4 and 4, vehicle- and THC-treated Glu-CB₁-RS, respectively; females *n* = 9 and 10, vehicle- and THC-treated CB₁-RS, respectively; 7 and 7, vehicle- and THC-treated CB_1_-STOP, respectively; 5 and 6, vehicle- and THC-treated GABA-CB₁-RS, respectively; 5 and 2, vehicle- and THC-treated Glu-CB₁-RS, respectively)]. *, p < 0.05 *versus* corresponding vehicle; ##, p < 0.01 *versus* *CB_1_-RS* vehicle group; ###, p < 0.001 *versus* *CB_1_-RS* vehicle group; $$$, p < 0.001 *versus* CB_1_-STOP vehicle group.
