# Supplementary figures and images for: Long-term hippocampal interneuronopathy drives sex-dimorphic spatial memory impairment induced by prenatal THC exposure
Source: Neuropsychopharmacology. 2020 Jan 26;45(5):877–86. doi: 10.1038/s41386-020-0621-3 (PMC7075920; doi:10.1038/s41386-020-0621-3)

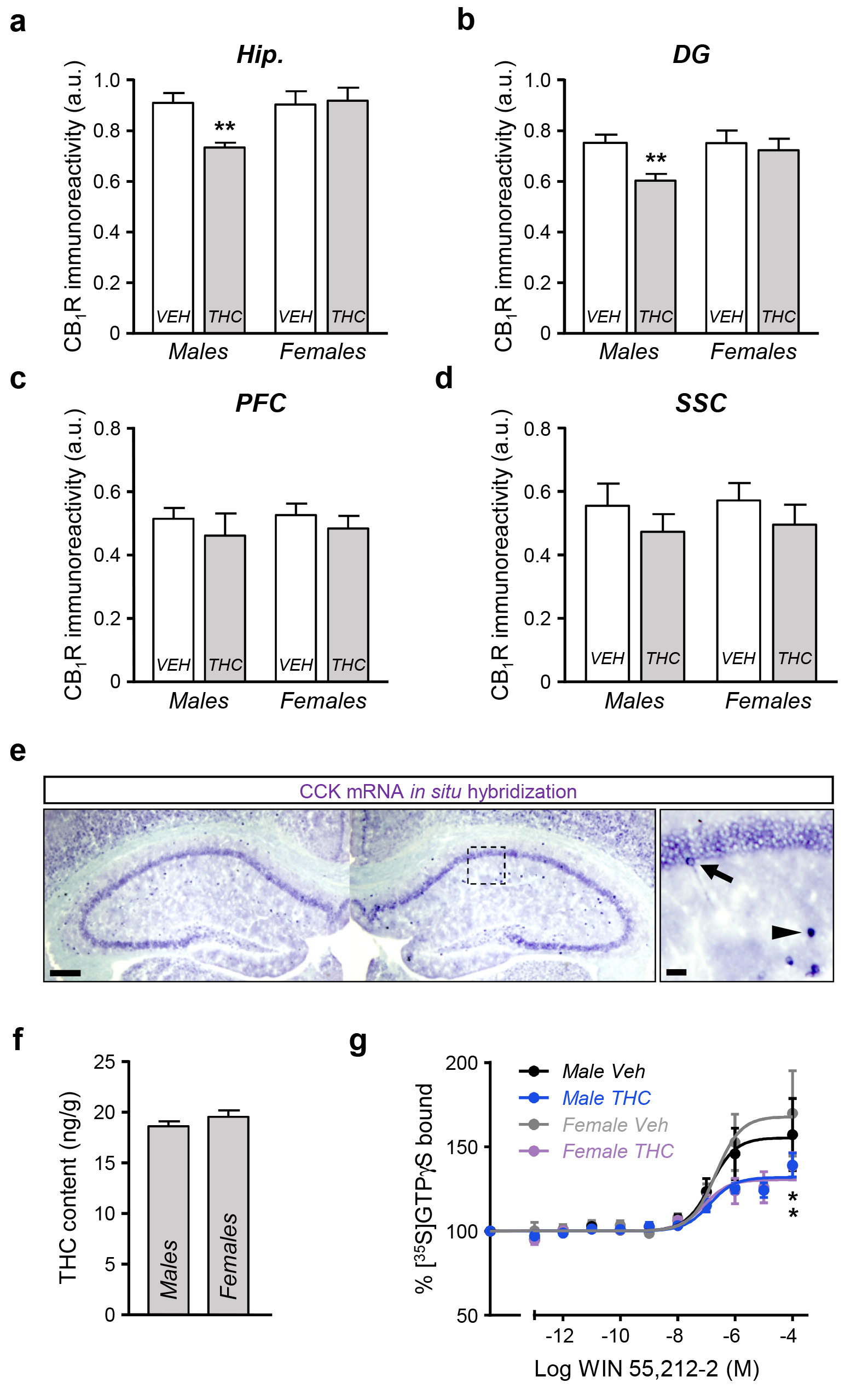

Supplement: Supplementary file 2 — Supplemental Material 1 [file 41386_2020_621_MOESM2_ESM.tif]

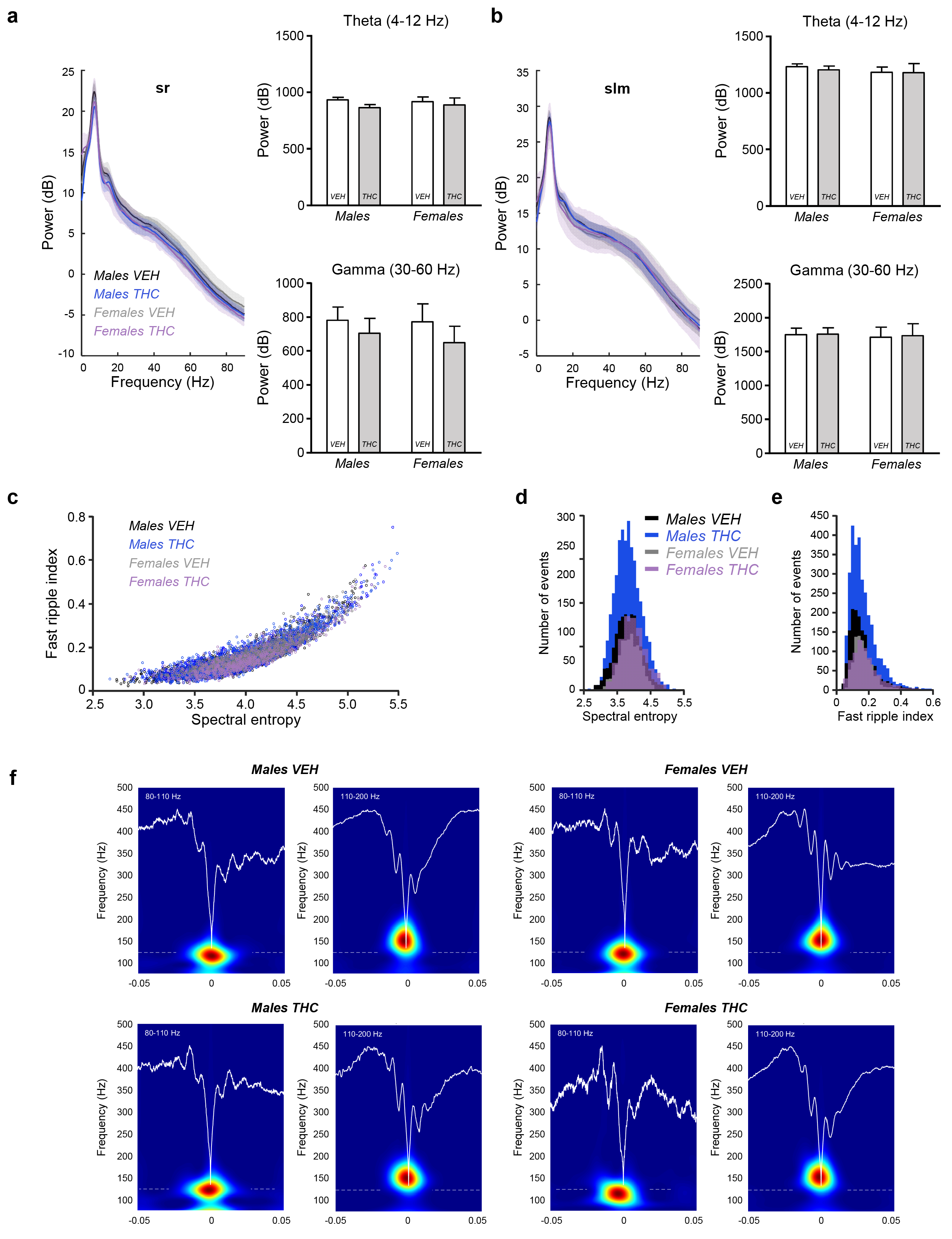

Supplement: Supplementary file 3 — Supplemental Material 2 [file 41386_2020_621_MOESM3_ESM.tif]

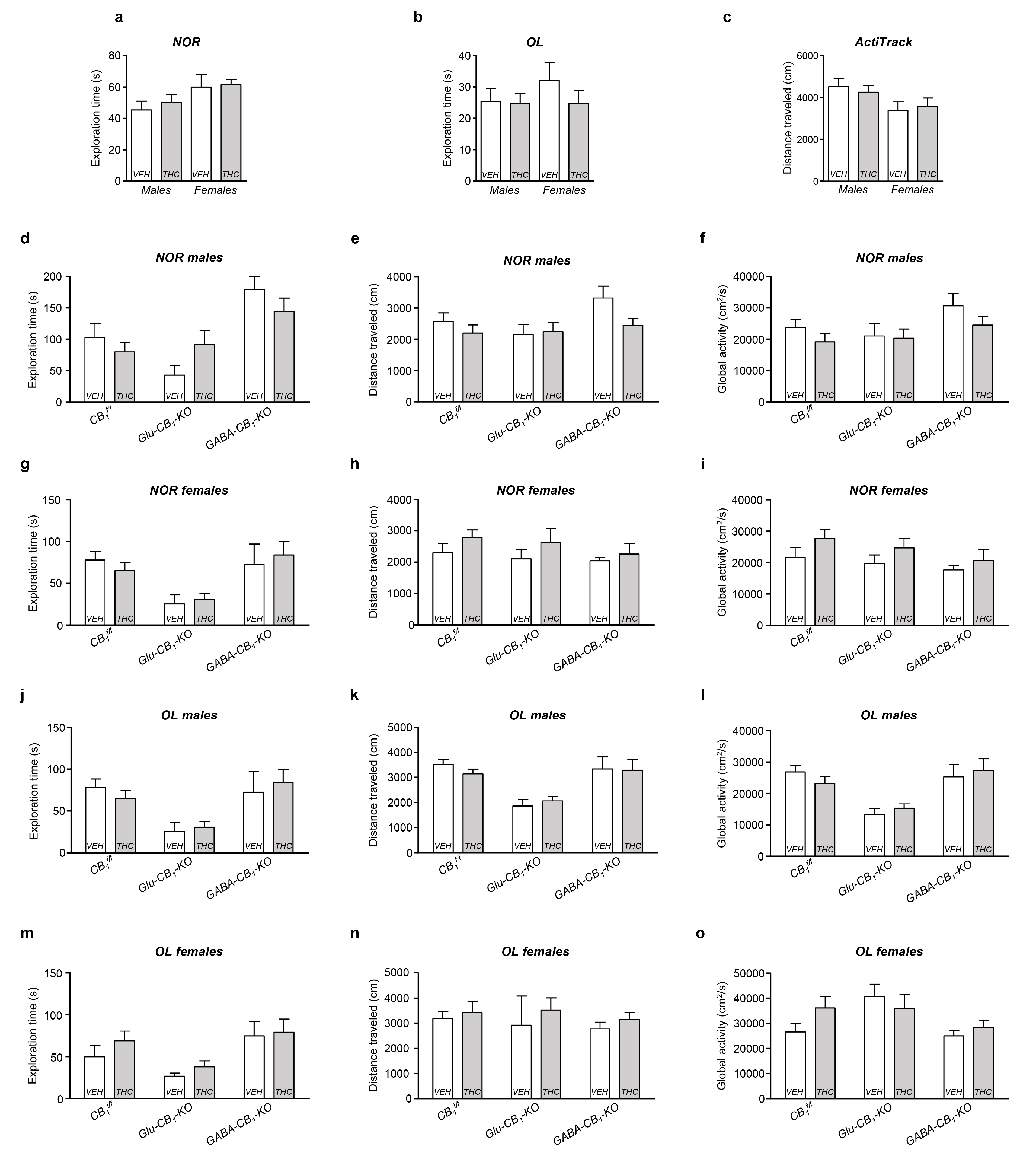

Supplement: Supplementary file 4 — Supplemental Material 3 [file 41386_2020_621_MOESM4_ESM.tif]

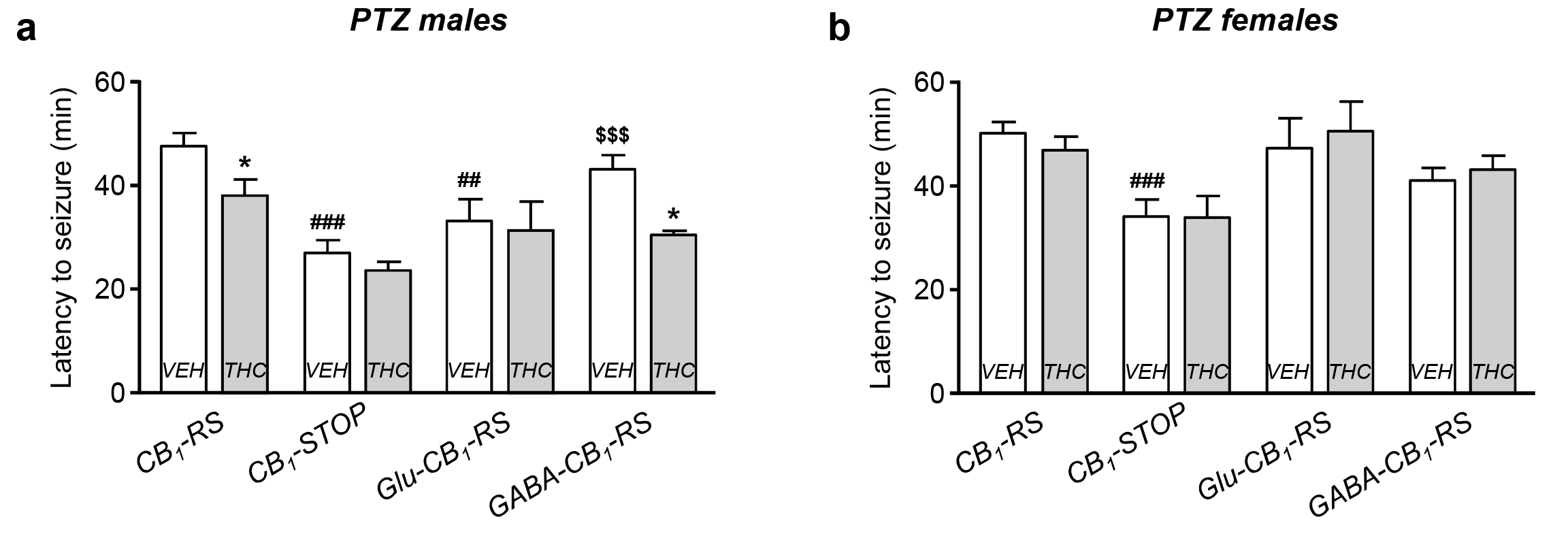

Supplement: Supplementary file 5 — Supplemental Material 4 [file 41386_2020_621_MOESM5_ESM.tif]
