# Supplementary material for: Long-term hippocampal interneuronopathy drives sex-dimorphic spatial memory impairment induced by prenatal THC exposure
Source: Neuropsychopharmacology. 2020 Jan 26;45(5):877–86. doi: 10.1038/s41386-020-0621-3 (PMC7075920; doi:10.1038/s41386-020-0621-3)
Supplement: Supplementary file 6 — Supp Methods [file 41386_2020_621_MOESM6_ESM.docx]

**Supplementary Materials and Methods**

**Animals**

Experimental designs and procedures were approved by the Complutense University Animal Research Committee in accordance with the European Commission regulations. All efforts were made to minimize the number of animals and their suffering throughout the experiments. Nex-CB_1_^-/-^ and Dlx5/6-CB_1_^-/-^ colony-founding mice were provided by Beat Lutz (Mainz, Germany). Mice were maintained in standard conditions, keeping littermates grouped in breeding cages, at a constant temperature (20±2 °C) on a 12h light/dark cycle with food and water *ad libitum*. The generation and genotyping of Nex-CB_1_^-/-^, Dlx5/6-CB_1_^-/-^ and wild-type (WT) littermates has been reported elsewhere, and was performed accordingly [1]. Mouse embryonic tissues were obtained upon timed mating as assessed by vaginal plug observation (E0.5).

**THC administration**

THC (≥99 % HPLC; THC Pharm, Frankfurt am Main, Germany) was diluted in 0.9% NaCl (saline) solution containing 3% (v/v) DMSO and 2% (v/v) Tween-80 and administered intraperitoneally (i.p.) at a final dose of 3 mg/kg to pregnant dams (at 10 µL/g) for 8 consecutive days, from E10.5 to E17.5. Control mice were injected with vehicle solution. There was no statistically significant difference in body weight or sex ratio between vehicle and THC-treated litters.

**Immunofluorescence and confocal microscopy**

Postnatal murine brain slices (30 µm-thick) were fixed in paraformaldehyde solution [4% in phosphate buffered saline (PBS)] at 4°C and immunofluorescence performed. After 2 h blockade with 5% goat serum, overnight incubation at 4°C with polyclonal guinea pig anti-CB_1_R (1:500, Frontier Institute, Japan) antibody was performed. Samples were subsequently incubated for 2h at room temperature with highly cross-adsorbed anti-guinea pig AlexaFluor 647 secondary antibody (1:500; Invitrogen, Carlsbad, CA, US). Confocal fluorescence images were acquired by using LAS-X software with a SP8 microscope, with 3 passes by Kalman filter and a 2048x2048 collection box. Immunofluorescence data were obtained in a blinded manner and sample code remained unsealed during the whole data processing and analysis. Immunoreactivity was measured using Fiji open source software establishing a threshold to measure only specific signal. The resulting binary mask was then used along the built-in measure function to acquire the total integrated grey density among all the pixels inside the binary mask overlaid on top of the original image. The obtained value was then referred to the number of DAPI^+^ cell nuclei present in the optic field.

***In situ* hybridization**

Coronal sections (30 µm) of P60 mouse brains were mounted on SuperFrost Ultra Plus glass slides (Thermo Fisher Scientific). When completely dry, they were subjected to *in situ* hybridization using CCK digoxygenin-labelled riboprobes obtained by PCR amplification and retrotranscription (forward primer 5’-AGAGAGCGGCGTATGTCTGT-3’, reverse primer 5’-CTGCATTGCACACTCTGGAC-3’). Briefly, samples were acetylated and then incubated in a hybridization buffer containing 0.5-1.0 µg/mL DIG-labelled riboprobes at 65 °C overnight. Sections were sequentially washed twice in a medium containing 2x standard saline citrate and 50% formamide for 1 hour at 60°C and then 20 min at 37°C in 2x SSC. The hybridized probe was detected after incubation with alkaline phosphatase-conjugated anti-DIG antibody (Roche) and subsequent incubation with NBT/BCIP (Roche) in alkaline buffer (pH 9.5). The resulting blue precipitate was post-fixed by incubation in PFA 4% during 10 min. There was no apparent signal in control sections with the sense probes.

**Immunohistochemistry**

Samples from ISH assays were subsequently used for IHC. Briefly, after washing, slides were subjected to a heat-induced antigen retrieval step with sodium citrate buffer (10 mM, pH 6) at 96ºC, as well as a 1 hour blockade, prior to exposure to a mouse monoclonal anti-GABA primary antibody (A0310; Sigma-Aldrich, Spain). Immunodetection was achieved using the Envision+ Dual Link System-HRP (DAB+) kit (Dako-Agilent Technologies, Santa Clara, CA, USA), following manufacturer’s instructions. Samples were then mounted using Entellan resin.

**Stereological analysis of cell density**

Brain sections stained by CCK-ISH and GABA-IHC were analysed using a Olympus BX61 microscope (Olympus, Tokyo, Japan) operated by VIS software with newCAST incorporated (Visiopharm, Hoersholm, Denmark). Hippocampal *strata* were manually delineated within CA1 region before performing a stereological counting of the total number of CCK^+^GABA^+^ cells. The frame area was set to 625 μm^2^ with a sampling interval of 120 μm at the z level. Results are expressed as number of immunoreactive cells per mm^2^. Gundersen’s coefficient of error was always below 0.1. Data were obtained in a blinded manner and sample code remained unsealed during the whole data processing and analysis.

**Pentylenetetrazol-induced seizures assay**

Pentylenetetrazol (PTZ) (Sigma-Aldrich Spain) was dissolved in 0.9% saline and administered i.p. to mice at P60 at a concentration of 22.5 mg/kg (at 10 µL/g). Mice were then placed in Plexiglas cages and re-injected with the PTZ-containing solution every 10 min until generalized seizures occurred. This was considered the end of the experiment. All the procedure was video-recorded and analyzed later by an experimenter blinded to the experimental groups, who determined the precise moment of generalized seizure onset.

**Novel object recognition task**

All tests were video-recorded and systematically analyzed by mean of the SMART 3.0 video tracking software (Panlab, Barcelona, Spain). P60 mice were habituated to an arena consisting on a grey methacrylate open field of 70 x 70 x 40 cm under uniform lightning conditions of 25 lux. Mice were allowed to freely explore the arena for 10 min the day before the novel object recognition (NOR) test to let them familiarize with the arena and minimize cage-exploring-derived distractions during the subsequent test. Test consisted of two parts: training and test sessions. During the former mice were allowed to individually explore two objects for 10 min. Then mice were returned to the home cage and the open field and objects were cleaned to avoid olfactory cues. After an inter-trial interval of 50 min, mice were placed back into the same open field for the test session, in which one of the familiar objects had been replaced by a novel object. Mice were allowed to explore the objects for 10 min. Mice did not show preference for any object before trials. Mice were excluded from the analysis only if the total time of exploration for each mouse was lower than 5 seconds in the test session. Object exploration was defined as the mouse being within 2 cm of an object, directing its nose at the object, and being involved in active exploration such as sniffing. A discrimination index (I_d_) was calculated to measure recognition memory as follows:

$$I_{d}= \frac{(t_{new object}- t_{familiar object})}{(t_{new object}+ t_{familiar object})}$$

**Object location task**

The day after NOR test, mice were subjected to an object-place recognition task in the same open field arena. This test was also structured in two sessions: similar to NOR, mice were first exposed to two identical objects that they were left to explore during 10 min. After that, mice were placed back in their home cage and the arena and object were cleaned to remove any odour cue. After 50 min, in the test session, one of the objects was placed in a different position maintaining its distance relative to the walls, and mice were allowed to explore the objects for an additional 10 min. Mice were excluded from the analysis only if the total time of exploration for each mouse was lower than 5 seconds in the test session. A discrimination index (I_d_) was calculated to measure recognition memory as follows:

$$I_{d}= \frac{(t_{displaced object}- t_{undisplaced object})}{(t_{displaced object}+ t_{undisplaced object})}$$

**Cannabinoid-induced analgesia and hypothermia**

THC-induced actions that depend on CB_1_R expressed in glutamatergic neurons but not in GABAergic neurons were evaluated in the P60 progeny of vehicle and THC-treated mice. Animals were injected i.p. with THC (10 mg/kg) or its vehicle, and basal temperature was measured with a rectal probe just before and 55 min after injection. Analgesia was determined 90 min after THC injection. Mice were placed in a hot plate set at 52ºC and the time until first sign of pain was quantified. In absence of signs of pain, mice were removed from the hot plate at a cut-off time of 30 seconds in order to avoid wounds to the animals. A naive cohort from each genotype was injected with vehicle and used to establish the baseline for both parameters.

**THC measurement**

Analysis of THC in embryonic brain tissue was performed as follows: samples were homogenized on ice with 700 μl of a mixture of 50 mM Tris-HCl buffer (pH 7.4): methanol (1:1) and the homogenates were transferred to 12 ml glass tubes. The homogenizer was washed twice with 0.9 ml of the same mixture, and the contents were combined into the tube with the homogenate. The homogenization process took less than 5 min per sample, and homogenates were kept on ice until organic extraction. After the homogenization step, 5 mL of hexane: ethyl acetate was added into each 12 mL glass tube and were placed in a rocking mixer over 20 min. Tubes were centrifuged at 1700 g over 5 min at room temperature and organic phase was then transferred to clean tubes, and evaporated under nitrogen stream. Extracts were subjected to a derivatization step using dansyl chloride. This reaction is selective for phenol functions present in THC and improves the separation and ionization efficiency. Extracts were reconstituted in 100 µl of a mixture of water:acetonitrile (10:90, v/v) with 0.1% formic acid (v/v) and transferred to HPLC vials. Twenty microliters were injected into an Agilent 6410 Triple Quadrupole Liquid-Chromatograph equipped with a 1200 series binary pump, a column oven and a cooled autosampler at 4 ºC (Agilent Technologies). Chromatographic separation was carried out with a Waters C18-CSH column (3.1 x 100 mm, 1.8 μm particle size; Waters, Milford, MA, USA) maintained at 40 ºC with a mobile phase flow rate of 0.4 ml/min. The composition of the mobile phase was: A: 0.1% (v/v) formic acid in water; B: 0.1% (v/v) formic acid in acetonitrile. THC was separated by gradient chromatography. The ion source was operated in the positive electrospray mode. The selective reaction monitoring mode was used for the analysis.

**WIN-55,212-2 stimulated [^35^S]-GTPγS binding assays**

Tissue samples were homogenized using a Teflon-glass grinder in 30 volumes of homogenization buffer (1 mM EGTA, 3 mM MgCl, 1 mM dithiothreitol (DTT), and 50 mM Tris-HCl, pH 7.4) supplemented with 250 mM sucrose. The crude homogenate was centrifuged for 5 min at 1000 g (4ºC). The resulting supernatant was centrifuged at the same conditions. Pellet was washed twice in 20 volumes of homogenization buffer and centrifuged in similar conditions. Binding assays were carried out in a final volume of 250 µL in 96 well plates, containing 1 mM EGTA, 3 mM MgCl_2_, 100 mM NaCl, 0.2 mM DTT, 50 μM guanosine 5′-diphosphate sodium salt (GDP), 50 mM Tris-HCl at pH 7.4 and 0.5 nM [35S]GTPγS (Perkin Elmer Life Sciences, Waltham, MA, USA). Stimulation curves were carried out by incubating increasing concentrations of the CB_1_ receptor agonist WIN 55,212-2 (10^-12^-10^-4^ M). The incubation started by addition of the membrane suspension (30 µg of membrane proteins per well) and was performed at 30ºC for 2 h with shaking (600 rpm). Incubations were terminated by rapid filtration under vacuum (Perkin Elmer Life Sciences) through GF/C glass fibre filters (Perkin Elmer Life Sciences) pre-soaked in ice-cold incubation buffer. The filters were then rinsed, air dried, and counted for radioactivity (5 min) by liquid scintillation spectrometry using a MicroBeta TriLux counter (Perkin Elmer Life Sciences). Non-specific binding was defined as the remaining [^35^S]GTPγS binding in the presence of 10 µM unlabelled GTPγS, and the basal binding as the signal in the absence of WIN55,212-2.

**Data and statistical analyses**

Results shown represent the means ± S.E.M., and the number of experiments is indicated in every case. Statistical analysis was performed with GraphPad Prism 6.07 (GraphPad Software, La Jolla, CA, USA). All variables were first tested for normality (Kolmogorov–Smirnov) and homoscedasticity (Levene’s). When variables satisfied these conditions, two-way ANOVA and LSD Fisher *post hoc* test were used to assess differences between groups. P values of <0.05 were regarded as statistically significant.

1. Monory K, Blaudzun H, Massa F, et al (2007) Genetic dissection of behavioural and autonomic effects of Delta(9)-tetrahydrocannabinol in mice. PLoS Biol 5:e269. https://doi.org/10.1371/journal.pbio.0050269
